# Genetic analysis of daily milk weights in US Holsteins using pen-based contemporary groups

**DOI:** 10.3168/jdsc.2024-0635

**Published:** 2025-03-01

**Authors:** Fiona L. Guinan, Robert H. Fourdraine, Francisco Peñagaricano, Kent A. Weigel

**Affiliations:** 1 Department of Animal and Dairy Sciences, University of Wisconsin–Madison, Madison, WI 53706; 2 Dairy Records Management Systems, Raleigh, NC 27603

## Abstract

The availability of daily milk weights and pen location information provides an interesting opportunity to capture additional data and review how contemporary groups are defined for dairy cattle genetic evaluations. In the United States, dairy cows in larger herds are grouped into pens according to various characteristics such as parity, production level, reproductive status, lactation stage, and health status. Our dataset included pen location information for each daily milk weight, so instead of using herd-year-season of calving to form contemporary groups, we used herd-pen-milking date to more precisely model the environmental effects cows experience at the pen level on a given day. Our dataset included 21,000,951 aggregated daily milk records from 114,243 first-parity Holstein cows milked 3 times daily in conventional parlor systems in 157 herds representing 29 US states. Our phenotype of interest was daily milk weight, and alternative repeatability animal models were used to estimate genetic parameters and predict breeding values. Age at first calving (6 levels) and DIM (10 levels) were included as fixed effects and cow (114,243 levels) was included as a random effect. Contemporary group effects included a fixed or random herd-year-season of calving effect (1,492 levels) and a fixed or random herd-pen-milking date effect (285,592 levels). Genetic parameters (kg^2^; posterior SD) were estimated using GIBBSF90+ software. The additive genetic variance ranged from 10.48 (0.60) to 24.12 (0.66), herd-year-season variance was 10.34 (0.40), herd-pen-milking date variance ranged from 4.91 (0.02) to 4.96 (0.02), permanent environmental variance ranged from 10.65 (0.44) to 16.94 (0.30), and residual variance ranged from 11.81 (0.01) to 14.60 (0.01). Heritability estimates ranged from 0.21 (0.01) to 0.47 (0.01), and repeatability estimates ranged from 0.51 (0.01) to 0.71 (0.01), and mean reliability of sires' breeding value predictions ranged from 0.81 to 0.89. Although caution is needed when disentangling associations between genetic effects, permanent environmental effects, and herd-pen-milking date contemporary groups, our results suggest that using daily milk weights and pen locations may improve the precision of genetic evaluations through increased sire PTA reliabilities for milk production traits in dairy cattle.

## Highlights

• High-frequency data in the form of daily milk weights are generated routinely on farms.
• Cows in large herds are managed in groups according to parity, health, and performance.
• Pen location information is valuable data to redefine contemporary groups.
• The use of high-frequency data can increase sire PTA reliabilities for production traits.

## Graphical Abstract

**Figure:**
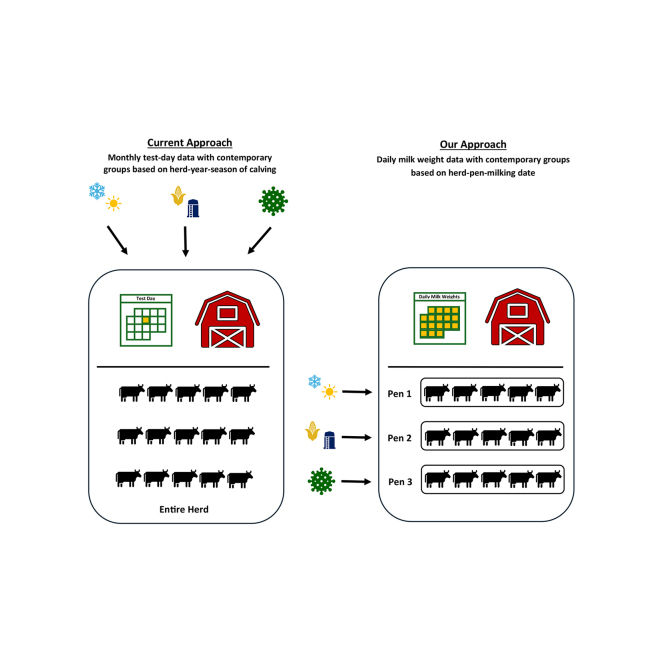
**Summary:** The advent of high-frequency data provides an opportunity to more precisely define contemporary groups for the genetic evaluation of milk production traits. Currently, in large herds, cows are managed at the group level rather than the herd level, which led us to investigate methods for incorporating these modern management effects into genetic evaluation models. We estimated genetic parameters based on daily milk weights, rather than traditional 305-day milk yields, and revised the definition of contemporary groups from herd-year-season to herd-pen-milking date. We found increased reliabilities of sire predicted transmitting ability (PTA) for production traits by including daily milk weights and updating the contemporary groups to reflect exactly where the cow was on a given date.

Precision livestock farming has advanced remarkably in recent years, generating extensive volumes of data (Lovarelli et al., 2020). The dairy industry has invested heavily in modern technologies to collect high-frequency data, monitor animals at the individual or group level, and inform labor decisions (Berckmans, 2017; Gargiulo et al., 2018). These high-frequency data are predominantly used for internal herd management purposes, whereas production and demographic data collected on test-day by milk recording organizations and averaged by on farm software are used in national genetic evaluation programs (Cole et al., 2021; National Dairy Herd Information Association, 2021). Daily milk weights are one example of high-throughput data, and other valuable information, such as the pen location of every cow, is also recorded at each milking in automatic milking systems or conventional milking parlors via electronic milk meters.

Contemporary groups for dairy cows are typically defined using the herd-year-season of calving when estimating variance components and predicting breeding values (Weigel et al., 2017). Therefore, each cow is assigned to only one contemporary group per lactation. This approach assumes that all cows calving in a given herd during the same year and season represent a cohort that experiences similar environmental conditions and management conditions throughout the lactation (Van Vleck, 1987). Given the unique nature of our novel dataset, our objective was to define contemporary groups more precisely and capture the actual or “micro-level” management and environmental conditions each cow experiences based on the pen she occupies on a given day. Additionally, it is possible that updating contemporary groups using within-herd grouping for other economically relevant traits such as fat and protein production that are currently not collected using sensor technologies could enhance genetic predictions. In the United States, cows in large herds are grouped in pens according to several factors, such as parity, milk production level, lactation stage, and reproductive status (Contreras-Govea et al., 2015). Managing cows at the pen level can improve labor efficiency, and cows in different pens within the same farm can be fed different rations to meet the nutritional needs associated with their physiological states (Barrientos-Blanco et al., 2022). The availability of daily milk yield phenotypes and assignment of pen-based contemporary groups can lead to a substantial increase in data availability, allowing for more accurate estimation of genetic parameters and increased reliability of sire PTA. As such, the aim of this study was to develop and evaluate models for genetic evaluation of dairy cattle that can fully use daily milk weights and pen locations. Four models were employed to assess the effect of changing the phenotype from 305-d milk yield to daily milk weights, examine the effects of modeling contemporary groups using herd-year-season of calving (**HYS**) or herd-pen-milking date (**HPM**), and assess the effect of treating them as fixed or random, on genetic parameters and reliabilities of sire PTA.

No human or animal subjects were used, so this analysis did not require approval by an Institutional Animal Care and Use Committee or Institutional Review Board. Data were provided by Dairy Records Management Systems (Raleigh, NC) and were extracted from the PCDART herd management software on their customers' farms. The 305-d milk yield (kg) and daily milk weights (kg) were extracted from PCDART. Detailed descriptions of the initial data edits can be found in Guinan et al. (2024). Additional edits include minimums of 25 cows per herd-year-season of calving, and 25 cows per HPM contemporary group. After the additional edits noted above, our dataset contained 21,000,951 daily milk weights of 114,243 primiparous cows milked 3 times per day in conventional parlor systems from 157 herds in 29 states. Four different models are described in Table 1, where AFC is the fixed effect of age at first calving (6 levels; ≤22, 23–24, 25–26, 27–28, 29–30, 30+), DIM is the fixed effect of days in milk (10 levels; 30 d each), HYS is the fixed or random effect of herd-year-season of calving (1,492 levels), HPM is the fixed or random effect of herd-pen-milking date (285,592 levels), cow is the random additive genetic effect using up to 5 generations of pedigree data distributed as $a∼\left(0,A\sigma_{a}^{2}\right),$ **pe** is the random permanent environmental effect distributed as $pe∼\left(0,I\sigma_{pe}^{2}\right),$ and **e** is the random residual effect distributed as $e∼\left(0,I\sigma_{e}^{2}\right).$ **A** is the numerator relationship matrix, and **I** is the identity matrix.

**Table 1:** Outline of the 4 different models used to estimate genetic parameters for 305-d milk yield (kg) and daily milk weights (kg)^1^

Model	
1	305-d milk (kg) = AFC + **HYS** + cow + e
2	Daily milk weight (kg) = AFC + DIM + **HYS** + cow + pe + e
3	Daily milk weight (kg) = AFC + DIM + **HPM** + cow + pe + e
4	Daily milk weight (kg) = AFC + DIM + **HYS** + **HPM** + cow + pe + e

1 AFC = age at first calving; HYS = herd-year-season of calving; HPM = herd-pen-milking date; pe = permanent environmental; e = residual. The contemporary group(s) for each model are in bold.

Model 1, which used 305-d milk yield (kg) as the phenotype and mimicked the Council on Dairy Cattle Breeding (Bowie, MD) national genetic evaluation system, served as a baseline for comparison with more complex models when estimating genetic parameters and predicting sire PTA. Models 2 to 4 used daily milk weights (kg) as the response variable. For models 1 to 3, the contemporary group (HYS or HPM) was fitted as either fixed or random, whereas for model 4, HYS was fitted as fixed and HPM was fitted as random. The GIBBSF90+ software was used to estimate variance components using a Bayesian approach employing Gibbs sampling with 50,000 samples. A total of 10,000 samples were discarded as burn-in, and 1 in 10 samples were stored to estimate posterior means and standard deviations (Misztal et al., 2024). Convergence was determined by visual inspection of the trace plots. Heritabilities (h^2^) were estimated using 2 formulas; h^2^ estimates include the contemporary group variance (when calculated) in the denominator, whereas h^2^* estimates do not include the contemporary group variance in the denominator. The PTA reliabilities (REL) were approximated using the following formula, $REL=1-\;\frac{PEV}{\sigma_{a}^{2}},$ where PEV is the prediction error variance calculated as the squared posterior standard deviation of the PTA estimate.

Estimated variance components from model 1 are not comparable with the others, due to a different scale when using 305-d milk production rather than daily milk weight as the phenotype. Depending on whether HYS was fitted as fixed or random, model 1 yielded estimates of 0.27 to 0.37 for h^2^ and 0.36 to 0.37 for h^2^*. For the remaining models, with the exception of model 3 when HPM was random, estimates of h^2^ ranged from 0.21 to 0.29 and estimates of h^2^* ranged from 0.27 to 0.29. The differences between model 1 and models 2 to 4 were expected, because the heritability of a daily milk weight phenotype is expected to be lower than that of a 305-d sum or average.

The primary objective of this research was to consider a pen-based definition of contemporary groups for analyzing daily milk weights by capitalizing on high-frequency data not currently used for national genetic evaluations. Currently, daily milk weights up to 10 d can be used to estimate 24-h DHIA test-day production if accurately labeled; however, these data are used solely to estimate milk production for test-day records (National Dairy Herd Information Association, 2021). Consequently, our novel contemporary groups were formed based on evolving factors that can affect the daily milk yield phenotype throughout the lactation period, including changes in ration, housing conditions, and other management practices. The effect HYS was used as a basis for comparison with both 305-d milk (model 1) and daily milk weights (model 2). For this section of results, we will focus primarily on comparisons of models 2 to 4. For model 2, depending on whether HYS was fitted as fixed or random, the additive genetic variance ranged from 10.76 to 10.85, contemporary group variance (when HYS was fitted as random) was 10.34, permanent environmental variance ranged from 15.01 to 15.08, and residual variance was 14.60. Model 3 gave similar results for variance components, with the exception of when HPM was fitted as random, which will be discussed in detail in the next section. Model 3 had a smaller residual variance than model 2, indicating that the environmental variance decreased when HPM was used as the contemporary group, as compared with HYS (Table 2). Finally, model 4 (HYS fixed; HPM random) had similar additive genetic variance (10.48) and permanent environmental variance (14.23) to models 2 and 3, with the exception of model 3 where HPM was fitted as random and had comparable residual variance with model 3 (11.85).

**Table 2:** Variance components, heritability and repeatability estimates (posterior SD), and sire PTA reliabilities for 305-d milk yield and daily milk yield using contemporary group (herd-year-season or herd-pen-milking date) as fixed or random effects^1^

Item	305-d yield (kg)		Daily milk yield (kg)				
	Model 1: Fixed	Model 1: Random	Model 2: Fixed	Model 2: Random	Model 3: Fixed	Model 3: Random	Model 4: Fixed and random
$\sigma_{cg}^{2}$	—	878,960 (33,617)	—	10.34 (0.40)	—	4.91 (0.02)	4.96 (0.02)
$\sigma_{a}^{2}$	837,300 (27,385)	842,500 (25,093)	10.76 (0.49)	10.85 (0.47)	11.96 (0.40)	24.12 (0.66)	10.48 (0.60)
$\sigma_{pe}^{2}$	—	—	15.08 (0.35)	15.01 (0.33)	16.94 (0.30)	10.65 (0.44)	14.23 (0.43)
$\sigma_{e}^{2}$	1,442,700 (20,438)	1,493,200 (19,145)	14.60	14.60	11.81	11.86	11.85
h^2^	0.37	0.27	0.27	0.21	0.29	0.47	0.25
h^2*^	0.37	0.36	0.27	0.27	0.29	0.52	0.29
*r*^2^	—	—	0.64	0.51	0.71	0.68	0.60

1 Depending on the model, contemporary group (cg) represents herd-year-season or herd-pen-milking date. $\sigma_{cg}^{2}$ = contemporary group variance; $\sigma_{a}^{2}$ = additive genetic variance; $\sigma_{pe}^{2}$ = permanent environmental variance; $\sigma_{e}^{2}$ = residual variance; h^2^* represents heritability calculated where cg is random without $\sigma_{cg}^{2}$ in the denominator of the h^2^ calculation; *r*^2^ = repeatability. Posterior SD for all estimates of $\sigma_{e}^{2},$ h^2^, h^2*^, and *r*^2^ were 0.01, with the exception of $\sigma_{e}^{2}$ for model 1.

For models 1 to 3, we also investigated differences between fitting the contemporary group (HYS or HPM) as fixed or random. The question of fitting contemporary groups to estimate genetic parameters as fixed or random is not novel (Schaeffer, 2009); however, we were interested in understanding the differences in variance component estimates, particularly when analyzing daily milk weights. Given the size of our dataset, specifically herd size, we expected to observe minimal differences among models. Additionally, as our dataset spanned only 5 yr, we did not anticipate a need to account for genetic trend or major improvements in management over time. For models 1 and 2, we found minimal differences between variance components when HYS was fitted as fixed or random. Interestingly, we found large differences between variance components when HPM was modeled as random in model 3. The estimates for residual variance did not change; however, the additive genetic variance increased from 11.96 to 24.12, whereas the permanent environment variance decreased from 16.94 to 10.65, indicating potential confounding between genetic and permanent environmental effects and difficulty disentangling these factors when treating HPM as random. In model 4, when HYS was fitted as fixed along with HPM as random, we found variance component estimates and heritability estimates (Table 2) that were comparable to those of models 2 and 3 (HPM fixed).

The large differences among variance components when treating HPM as fixed versus random in model 3 were unexpected. Our hypothesis is that short- and medium-term management and environmental effects common to cows within a given HPM contemporary group created confounding between HPM effects and permanent environmental effects when using daily records. This confounding, in turn, caused instability in the variance component estimates for additive genetic effects. As well as that, grouping decisions are designed to allow homogeneous management groups of cows with similar physiology, nutritional needs, and performance, and thereby avoid labor associated with managing large numbers of cows individually. In many cases, highly overlapping groups of cows were in the same pen for many consecutive days or weeks, and these short- and medium-term correlations among management and environmental effects could not be easily parsed into temporary (i.e., residual) and permanent environmental effects. Additionally, the manner in which farmers make grouping decisions can create a risk of confounding between the genetic effects, contemporary group effects, and permanent environmental effects. Cows within each herd are reallocated to HPM groups throughout the lactation, as their milk production, pregnancy status, health, and body condition change. In some cases, pen assignments for upcoming days or weeks are made using data regarding daily milk yield phenotypes expressed in the most recent days by individual animals and their herdmates, creating the potential for confounding between genetic effects and contemporary groups. In traditional analyses of 305-d milk production, contemporary groups were assigned based on herd, calving year, and calving season for each cow, and all of these variables were observed before the milk yield phenotype was recorded. Therefore, we developed model 4 as a potential solution, in which HYS was included as a fixed effect alongside HPM as a random effect, and this adjustment provided estimated genetic parameters that were comparable to previous models and more consistent with our expectations based on the scientific literature. Therefore, we believe adding a fixed HYS effect can help reduce the confounding between genetic and permanent environmental effects associated with repeated daily milk yield records of cows in the same pen, while allowing the added precision of a pen-based contemporary group definition.

Sire PTA reliabilities were estimated to assess whether using large volumes of daily milk yield data and assigning contemporary groups using pen information would increase the accuracy of selection decisions. We found a 0.02 increase in mean REL of sire PTA when using daily milk weights as the phenotype in comparison to 305-d milk yield (Figure 1). Aside from model 3, when HPM was fitted as random, we did not observe differences among reliabilities when fitting contemporary group as fixed or random (Figure 1). We did not observe differences in sire PTA reliabilities when using HPM as the contemporary group instead of HYS, although one should note that these REL values are approximations, and assessments using alternative approaches such as cross-validation might be more precise. In addition, it is possible that employing an autoregressive error structure to account for correlations among residuals within HPM levels might be beneficial in terms of the reliability of predicted breeding values.

**Figure 1:**
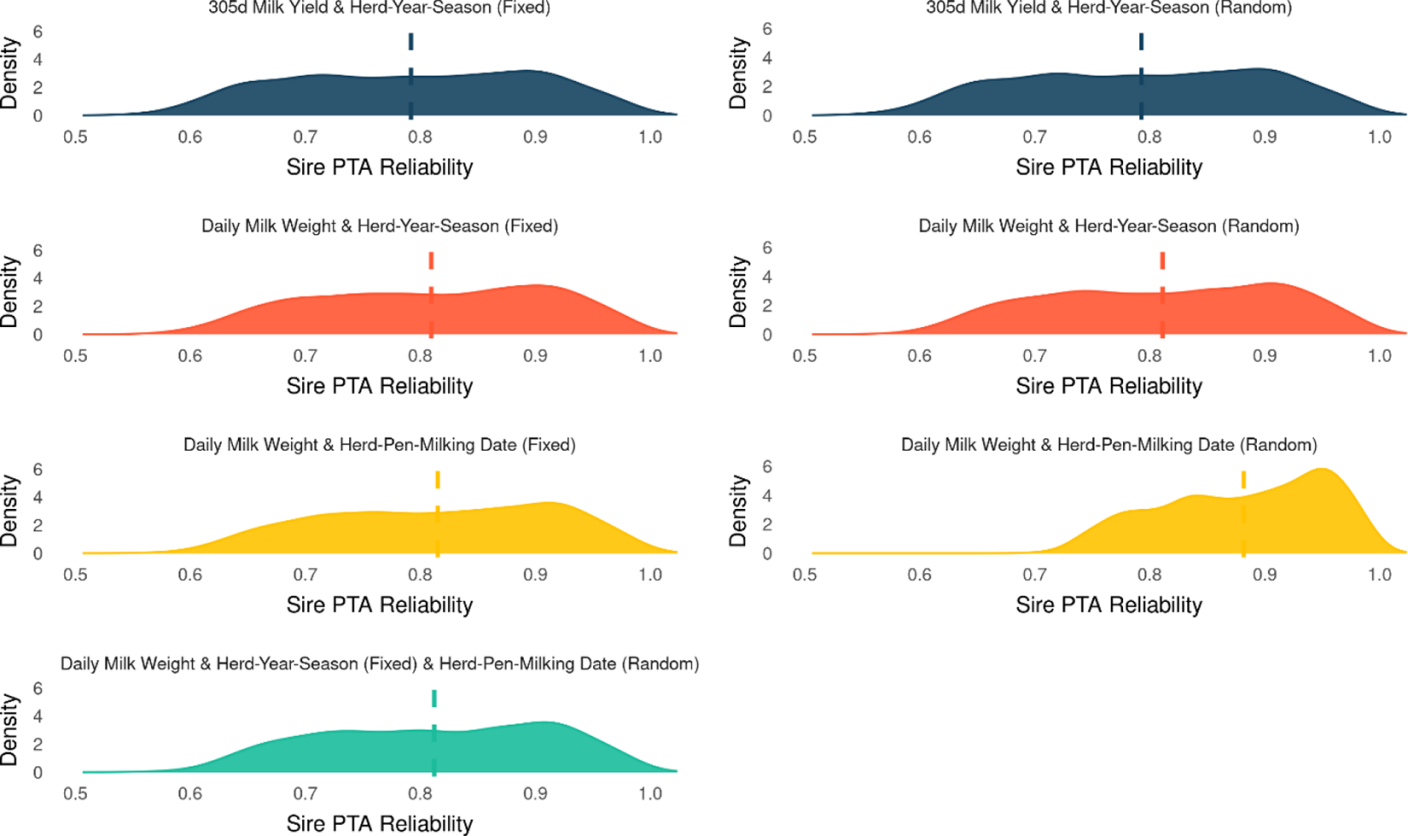
Distribution of 1,620 sire PTA reliabilities with ≥10 daughters when contemporary group is fitted as fixed or random (models 1–3), or both fixed and random (model 4). The vertical dotted lines represent the mean sire PTA reliability for each individual model.

The advent of high-frequency novel data sources for use in genetic evaluations of farm animals creates opportunities but may require new definitions for contemporary groups. In the specific case of milk production, the reliabilities of sire PTA increased when using daily milk weights as opposed to 305-d milk production. Changing the definition of contemporary groups from HYS to HPM affects genetic parameter estimates and sire PTA reliabilities, as does the decision of treating contemporary groups as fixed or random. Future research on optimizing models for estimating genetic parameters and predicting breeding values using high-frequency data generated by sensors should focus on strategies for creating contemporary groups precisely while mitigating the potential for confounding with genetic and permanent environmental effects. In our case, including HYS as a fixed effect along with HPM as a random effect seemed to accommodate the challenge of shared relationships among residuals for cows within the same pen over adjacent days or weeks. In summary, the widespread adoption of precision livestock farming technologies on large dairy farms provides high-frequency phenotypes, as well as day-to-day information that can be used to model management and environmental conditions, and strategies for incorporating these data into national genetic evaluation systems are proposed in this analysis.
